# Teaching Oncology in the Medical Degree: Integrating Knowledge and Experience of a Multidisciplinary Tumour Board

**DOI:** 10.1007/s13187-025-02784-y

**Published:** 2025-11-18

**Authors:** José Expósito-Hernández, Mariana F. Fernández, Mercedes Villalobos, Javier López-Hidalgo, Rosario Guerrero-Tejada, Isabel Tovar-Martín, Rosario Ching-López

**Affiliations:** 1 Radiation Oncology Department, Virgen de Las Nieves University Hospital, Granada, Spain; 2 Biomedical Research Institute (Ibs.GRANADA), Granada, Spain; 3 Radiology and Physical Medicine Department, Faculty of Medicine, University of Granada, Granada, Spain; 4 Consortium for Biomedical Research in Epidemiology and Public Health (CIBERESP), Madrid, Spain; 5 Pathological Anatomy Unit, San Cecilio University Hospital, Granada, Spain

**Keywords:** Medical school, Oncology education, Knowledge integration and multidisciplinary tumour board, Team-based learning, Flipped classroom

## Abstract

Cancer is recognised as a major health problem that requires a multidisciplinary approach to achieve the best possible outcomes. The rapid and important changes in the understanding of cancer need to be incorporated into clinical practice. Because of both the magnitude and pace of these changes, medical schools must also assess and update the level of knowledge and skills that need to be taught in oncology training. Multidisciplinary tumour boards (MTB) integrate the knowledge of various healthcare disciplines, assuring treatment according to the best available evidence or multidisciplinary consensus. However, these attributes are not sufficiently integrated into the curricula of Spanish medical schools. To fill this gap, specific seminars were designed and implemented during the medical degree programme at the University of Granada (Spain), over two academic years for 510 students. The first group focused on integrating knowledge in the field of oncology, while the second group focused on the purpose of the MTB in clinical practice and its role in decision-making in specific tumour cases. All the seminars included real cases of patients with medium-complexity tumours, along with the clinical practice guidelines from a university hospital as a reference. After two years of development, the initiative has been evaluated (15-items survey), with a high level of satisfaction among students and teachers, but awaiting the collection of objective learning outcomes. This article describes the seminars, along with how they were organized. and discusses the value of introducing validated pedagogical techniques. The incorporation of well-received pedagogical innovations, such as team-based and small-group teaching, together with ‘flipped classroom’ techniques, focuses both on students’ individual knowledge and ability to interact with others.

**Supplementary Information:**

The online version contains supplementary material available at https://doi.org/10.1007/s13187-025-02784-y.

## Introduction

**Fig. 1:**
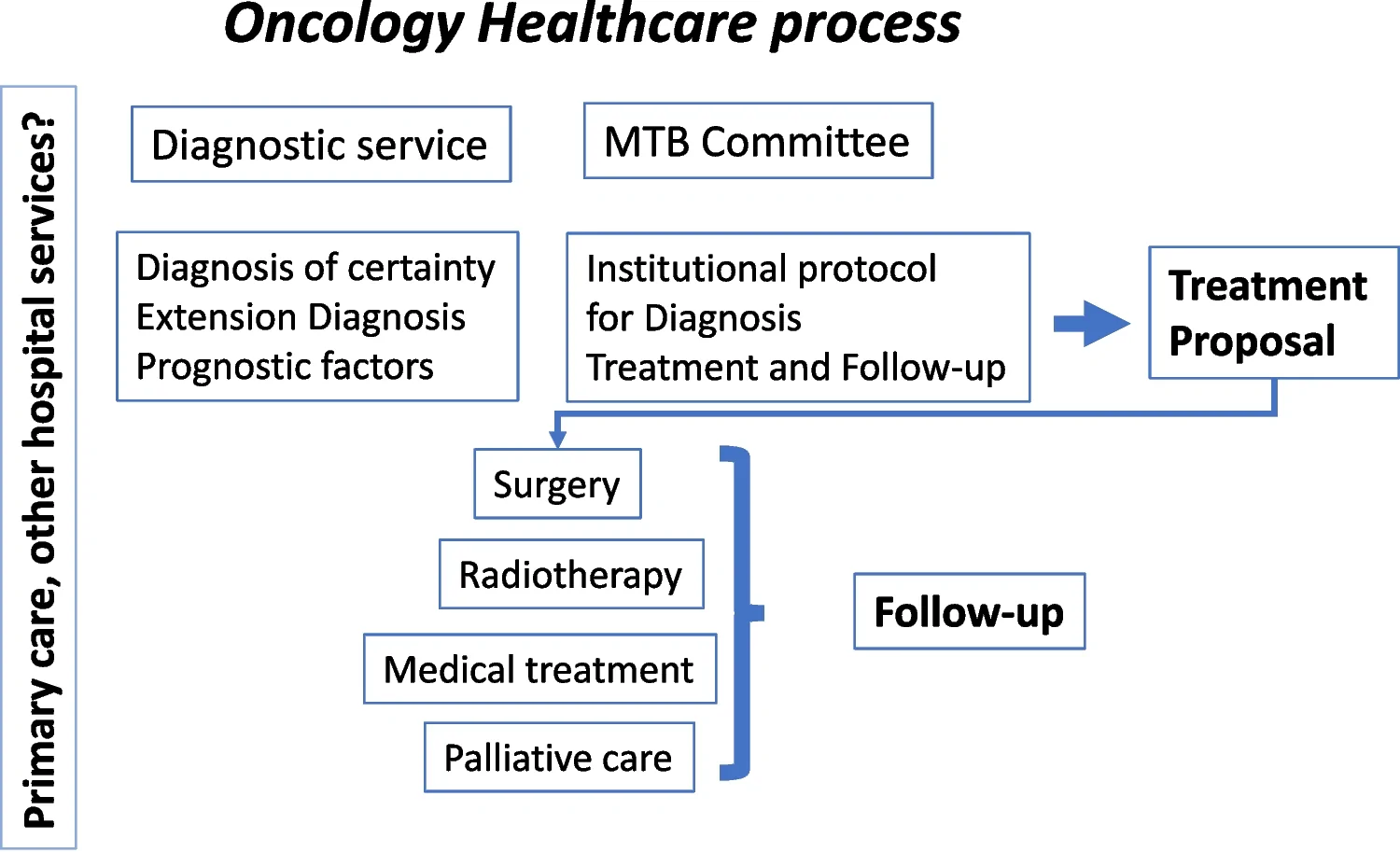
When a malignant tumour is suspected, the first care service, determined by the patient’s clinical presentation (diagnosis service), must obtain a definitive diagnosis, establish the extent of the disease and assess prognostic factors. With this information, it is possible to deduce a clinical classification of the case, which is then discussed in the corresponding multidisciplinary tumour board. Ideally, the committee should agree on the development of a complete treatment plan, in which the different steps appropriate for that patient are ordered, based on the current consensus protocol in the hospital

Cancer is recognised as a major health problem, both in terms of frequency and social and health costs. In the comprehensive care of cancer, it is well stablished that effective clinical management at all stages of the disease impacts the results and expectations of the patient [1]. It should also be borne in mind that the knowledge surrounding the tumour process (natural history, prevention, screening, diagnosis and treatment, etc.) is undergoing continuous and rapid changes, with a positive impact on overall outcomes [2]. The current care process around the cancer patient is summarised in Fig. 1.

The aforementioned characteristics (magnitude and accelerated/rapid change) often lead medical schools to consider what level of knowledge and skills should be taught in oncology education, considering that good training of future professionals will also have an impact on long-term outcomes. In recent years, many academic fields have developed new teaching proposals [3, 4], with the following common elements: i) specific curricula underlining/emphasising the value of knowledge integration, and ii) multidisciplinary education, embodied in the clinical model of the MTB, as the most efficient tool for patient care management [5], and iii) pedagogically effective learning strategies.

The main objective of the teaching proposal focuses on the need to integrate knowledge and offer interdisciplinary learning based on real clinical cases with the working approach of a multidisciplinary tumour committee. Both aspects are essential in today’s oncology clinics and must be taken into account by any future healthcare professional. In fact, these learning objectives are explicitly recognised in the main leading training programmes, which highlight teamwork and specific areas such as MTB, as essential areas of knowledge in the oncology curriculum for university students [3, 5].

However, many medical schools/faculties do not have a specific oncology programme in their curricula, and teaching about cancer tends to be heterogeneous, fragmented, and lacks adequate interdisciplinary reflection in its content. On the contrary, contents tend to be essentially adapted to the curricular areas of the departments involved, without a formal assessment of their weight throughout the teaching of the current medical degree (epidemiological, basic, therapeutic, palliative, etc.) [6].

In particular, the teaching of oncology at the medical school of the University of Granada (Granada, Spain), as in many other institutions, suffers from these drawbacks, and provides a very ‘departmentalised’ curriculum, focused on basic sciences, with few periods of clinical teaching (mostly practical), and lacking integrated interdepartmental activities [7]. Thus, training in oncology is delivered on a system-by-system basis, with the difficulties that this entails for learning the general principles of pathobiology. Nevertheless, the teaching effort devoted to oncology (in terms of the number of credits-hours and total numbers of subjects) is considerable (174 specific credits spread over 15 subjects over the 6 years) (Teaching guide, 2024–2025) [8]. This teaching activity does not take into account whether or not new pedagogical techniques are introduced in the field of medical education [9], nor does it take into account the periods of practical assistance.

To improve the teaching of oncology and address the aforementioned shortcomings, teaching seminars were designed in line with to the new academic recommendations, and the guidelines of scientific societies [10, 11] (Supplementary Table 1). As a result, two parallel study forums were conducted among 510 undergraduate students during two consecutive academic years, with the aim of integrating knowledge through a multidisciplinary approach, and based on the incorporation of well-appreciated teaching innovations [12]. The initiative stems from an awarded teaching innovation project (TIP) [13]. Both activities are presented and discussed below.

## Development of Specific Seminars

During the development of the TIP, two types of compulsory seminars were designed, taking into account the current oncology clinical care model, within the oncology module of the radiology and physical medicine course, for students in their fourth year of the Medicine bachelor’s degree at the University of Granada (Spain). The radiology and physical medicine subject, with 10 teaching credits, includes radiobiology, radioprotection, radiology, rehabilitation, nuclear medicine and radiation oncology. It is taught after students have already completed basic subjects such as histology, pathological anatomy, molecular biology, immunology, biochemistry, and pharmacology. The seminars are organised in the second semester of the academic course, when students have already received more specific training on lung cancer and digestive and urological tumours (digestive, pulmonary and genitourinary pathology subjects), but with little or no contact with clinical oncology practice.

### 1st. Knowledge Integration Seminar

#### Objective

**Fig. 2:**
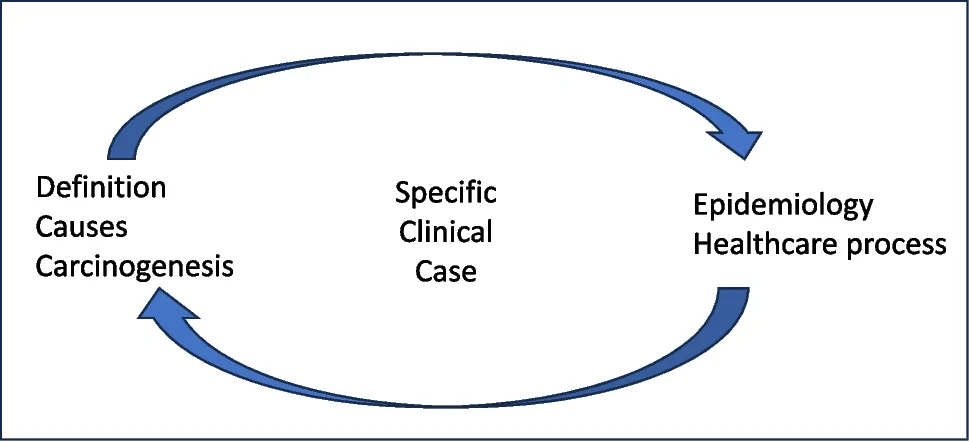
Seminar outline (knowledge integration)

To review, through a circular approach, what has already been learned about cancer, following the ‘clinical case’ model (Fig. 2); presentation of information using epidemiological and clinical data (incidence, mortality, environmental exposure, etc.). The frequency was weekly, for two hours and in small groups (20–24 students).

### Dynamics of the First Seminar and Operational Procedure

a. The previous week, students receive, through the teaching platform (PRADO), information on a clinical case, with a complete clinical history, diagnostic tests, staging (size and location, localised or dispersed), treatment information and clinical evolution.

b. On the day of the seminar, the teacher presents the objectives of the seminar and summarises the clinical history. The lecturer stops after the staging investigation has been completed, before the MTB’s treatment proposal, i.e. at the end of the Diagnostic Service phase of Fig. 1 (30 min).

c. The lecturer then elaborates on the overall oncological process of the clinical case, including conceptual aspects of aetiology, risk factors, carcinogenesis, cellular kinetics (early diagnosis) and epidemiology, already covered in other subjects (45 min). Special attention is given to the concepts of cancer as a preventable disease and the European Code Against Cancer [14], as well as the role of cancer population registries. The discussion of these concepts allows linking risk factors and exposure to the specific development of a neoplasm.

d. Next, attention is focused on the complete treatment received by the patient, as a result of the discussion of the case in the MTB, focusing especially on the radiotherapy treatment (radiotherapy workflow: simulation, treatment planning, dose-volume histograms, etc.), as well as on the clinical evolution, considering and analysing other possible risk and exposure factors (30 min).

e. Final summary, analysing the entire process of patient care, possible delays, as well as the outcomes in terms of survival, toxicity and quality of life (15 min).

Before the end of the seminar, the students prepare, in groups: i) a summary of the 10 essential ideas incorporated (‘take-home’ points), ii) an evaluation of the process the patient has undergone, and iii) some questions (3–4) raised by the information from the seminar. These last three items (summary, evaluation of the process and questions) are reviewed by the teacher according to four criteria [a) preparation/organisation, b) content, c) clarity/coherence, and d) conclusion], and are individually graded as excellent (exceeds expectations), good (meets expectations) or needs improvement. The same seminar is repeated weekly so that all students enrolled in the 4th year attend. The total duration of the session is 2 h.

### 2nd. Interdisciplinary Oncology Seminar

#### Objective

To become familiar with the work (and discussion) of the MTB, an essential forum for decision-making in oncology, based on the interaction and consensus among experts, and on current work in cancer care. From this perspective, the distribution of students and the content of the information they receive are designed, incorporating the comments of each of the experts participating in the debate on the case-study on which the seminar focuses.

Students receive in advance the complete clinical case of a patient with a specific tumour, in addition to the complete hospital protocol in force for that type of cancer.

**Fig. 3:**
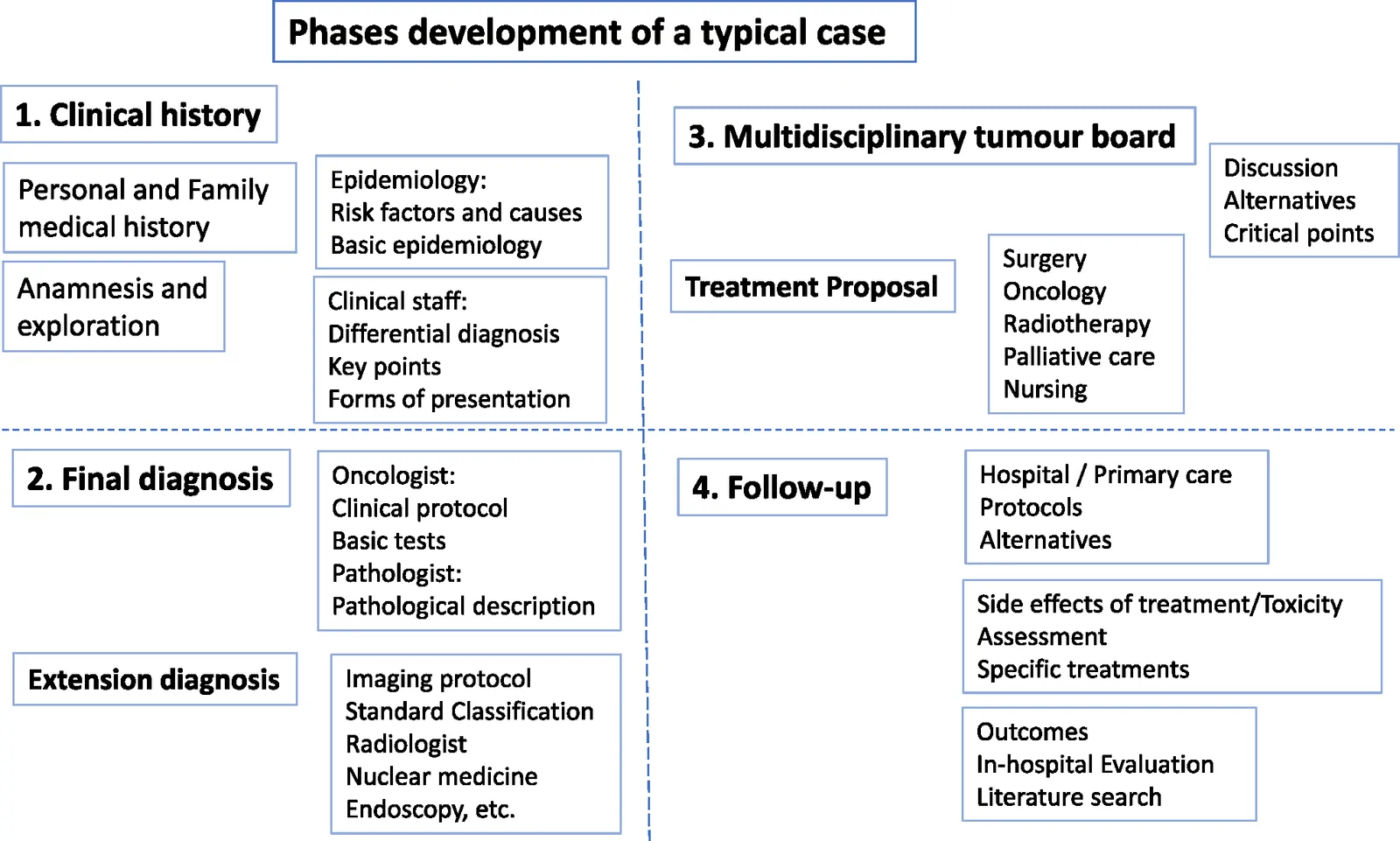
Clinical case development in 4 phases

A total of 12 clinical cases of patients with prevalent tumour locations of medium difficulty and complexity were elaborated (see examples in the supplementary material). The general outline of the oncological care process (Fig. 1) was followed, divided into four phases (Fig. 3). Each case included the report drawn up by the relevant experts: pathologist, radiologist, nuclear medicine, specialist, etc.

**Phase 1. Complete medical history and clinical examination**. This includes the complete anamnesis and clinical examination, as well as three questions oriented to the data of the anamnesis, examination, or differential diagnosis, which will be discussed internally by the subgroup of students acting as the MTB. Additional guiding comments of particular interest may also be included (see an example of prostate cancer in the supplementary material).

**Phase 2. Diagnosis of certainty (histological study) and of extension (imaging and analytical tests)**. This phase allows consideration of the necessary tests (and their results). It allows the inclusion of the most representative imaging tests, selected by experts, with comments and questions (see an example of lung cancer in the supplementary material).

**Phase 3. Discussion in the MTB and proposed treatment** (presented by the expert with the greatest responsibility for the case). In this phase, more detailed information can also be presented, for example, the radiotherapy treatment, incorporating images of the simulation, volume design, planning and dose-volume histogram. The expert’s comments also complete the information needed to evaluate the questions raised (see an example of breast cancer in the supplementary material).

**Phase 4. Follow-up of the case, expected side effects and results.** In this phase, it is very important to introduce and raise awareness students of common recommendations for patients. Comments and questions are again included. This stage also allows for the introduction of other treatment modalities for cancer patients (integrative oncology).

In summary, each phase includes information along with relevant contributions and comments from expert specialists. Each of the four phases concludes with several questions (3–4) proportional to the knowledge acquired, which must be answered by the group of students (Fig. 3).

### Dynamics of the Second Seminar and Operational Procedure

a. Students receive the complete clinical information and the active protocol for the specific tumour site the week before via the teaching platform (PRADO).

b. On the day of the seminar, a brief presentation of the case is given by the lecturer and the students are divided into groups of 5–6 (similar to the MTB) (30 min).

c. The groups of students discuss each case from phase 1 to phase 4, and answer the questions raised (45 min).

d. Answers and possible disagreements between the different subgroups are shared (30 min).

e. The seminar ends with a summary by the trainer including: i) specific comments on aspects of the treatment, ii) brief description of integrative strategies that could have been used, and iii) references to environmental exposures and cancer (15 min).

This seminar is led by a clinical oncologist who uses reports and comments provided by other expert clinicians (pathologists, radiologists, nuclear medicine specialists, etc.) on each clinical case (biochemistry, imaging, etc.). Students are also informed of the discussion that took place during the presentation of the real case at the tumour board. In this way, they are present in the committee simulation. Importance is also given to the answers that the group of 5–6 students gives for the questions asked. At the end of the seminar, a discussion is held about the different answers given by the different groups, with the teacher providing any necessary clarifications. The total duration of the session is 2 h.

## Evaluation and Outcomes of the Seminars

For the evaluation of the first seminar, the results and comparison of the three final items (summary of 10 key points, evaluation of the patient process, and questions generated by the seminar) were taken into account.

For the evaluation of the second seminar, a Google survey (Google, Alphabet Inc., Mountain View, USA) with 15 questions (answers scored from 0 to 10) was used (included in supplementary materials). This no-validated questionnaire was developed by the working team as a first approach to evaluating student reaction/satisfaction and, to a lesser extent, knowledge acquisition [15], with questions addressing both the quality of the information and the seminar format, as well as the role of the MTB and student involvement. It was completed by 383 students (75% response rate), with average scores ranging from 75 to 92 out of 100. Overall, the highest scores were obtained in questions 8, 9, and 10, indicating a strong understanding among students. It was particularly noteworthy that their level of participation was also considered high (91% with high score), as well as the role of the MTB in the seminar´s development (89% with high values). Additionally, 79% of the students rated them as appropriate for their training (see Supplementary Table 2).

A global review of the seminars was finally conducted with the experts and lecturers (discussion/focus group) involved in the seminar design and case selection to identify possible areas for improvement. This process led to enhancements in the information provided (see the final assessment questionnaire in the supplementary material) and in the evaluation of objective learning outcomes for subsequent academic courses. It was also decided to include several multiple-choice questions aligned to the stated seminar´s objectives in the overall evaluation of the Radiology and Physical Medicine subject.

## Discussion

The development of the above teaching seminars, designed to improve the teaching of oncology, facilitates medical students’ learning through pedagogically appropriate teaching techniques. In addition, they address two key current aspects: the integration of knowledge and the multidisciplinary approach to multifactorial diseases. The results of the evaluation and the perception of the students (and teachers), after being conducted during two consecutive academic years (2022–23 and 2023–24), in which a total of 510 students participated, seem to indicate that the new teaching strategy has been successful.

The teaching of oncology in the medical degree program has been subject to various evaluations and criticisms, which have affected both its theoretical and practical teaching [9]. Most of these highlight the need for formal presentations of topics (didactic readings) to be complemented with validated techniques that are less passive and more student-centred [12]. The seminar experience described here introduces some of these techniques. It incorporates aspects of problem-based learning methodology along with its resolution, using and presenting clinical scenarios that gather clinical information and updated protocols (as well as possible alternatives) to which the student has access. The design also adds work dynamics that take into account both team-based and small-group teaching, as well as flipped classroom techniques [16–18], where the essential focus is on the student’s effort and their capacity for interaction.

In addition to “team learning”, other validated teaching experiences were also taken into account in the organisation of the seminars. This includes “problem-based learning”, with real and active student participation in the learning process. To this end, the seminar begins with a proposal based on a specific clinical case, which students must first study outside the classroom. Problem-based learning has recognised educational advantages [16]. In this project, the underlying idea is that through the information provided in the clinical case and its uncertainties, students will understand related health problems, as well as the basic structure of cancer patient care in its different phases (diagnosis, discussion and application of treatment, toxicity and final outcomes).

The seminars fit in with the flipped classroom concept, as information is available in advance and participants can prepare beforehand, allowing them to spend the seminar time reinforcing the knowledge needed to understand the subject matter (integration seminar) and any subsequent problems that may arise in the different stages of the care process (MTB seminar). In addition, the face-to-face seminar-based learning is carried out in small groups (between 5 and 6 students) who roleplay as experts in a tumour committee. They follow the clinical case script together with questions to be resolved that explore different possible alternatives, both in terms of the interpretation of signs and symptoms and the value of the different therapeutic alternatives [9, 17]. Teaching improvement is further assessed through self-evaluation of knowledge and satisfaction levels, both from students (surveys) and professors (focus group).

The positive aspects of this experience include i) its ease/feasibility of incorporation into the academic calendar without adding more theoretical teaching burden on the students, ii) the integration of knowledge that the student has already received in other subjects, iii) the collaboration and participation of different specialists, enabling a multidisciplinary approach to teaching oncology, and iv) its ethical approval by the FIDO (acronym in Spanish for Training, Innovation and Teaching Evaluation) programme at the University of Granada.

The current predominant model for teaching oncology in medical schools is based on the study of pathology by organs and systems [17]. However, this pilot experience could lead to an alternative or complementary learning approach in a future Integral Oncology course. A similar learning model would contribute to a new structure for knowledge and subjects in the medical degree program/curriculum, providing a better balance in the contribution of different experts/specialists, beyond the mere exchange of credits that currently exists between departments. Furthermore, greater coordination of the teaching plan is essential, allowing, among other things, clinical cases to cover a wider range of tumour locations and clinical situations.

However, there are many points for improvement in the design of the described seminars. This includes increasing the number and variability of the clinical cases currently included and incorporating patients in different clinical situations (patients eligible for palliative treatments, where decisions are usually more complex, patients eligible for high-tech therapeutic approaches, such as surgical techniques, high-precision radiotherapy, or CAR-T treatments, etc.). Among the shortcomings of this project, there was no formal evaluation of the results, both in terms of knowledge acquisition and skill development by students, through a validated system. This could include pre- and post-learning tests or clinical reasoning assessments, which would facilitate the measurement of the effectiveness of the proposed teaching method. Moreover, we consider it highly valuable to introduce other open teaching spaces with the active involvement of patient associations, as well as other “new” actors who are already essential in the current treatment of oncology patients. This includes appropriate physical activity, diet and healthy lifestyle habits, reduction of environmental chemical exposure, or emotional support through psychological assistance, among others [19, 20]. As more evidence is generated in this field, it will be essential for healthcare professionals to be aware of the prevalent use of these new modalities of integrative oncology. Some programmes, such as Integrative Oncology Scholars (IOS) already enable students to improve their skills, allowing them to include, discuss and/or recommend these evidence-based integrative therapies alongside conventional cancer treatments [20]. It is therefore worth considering whether integrative oncology education programs are not only suitable but also essential.

### Final Observations

The development of case-based seminars, grounded in the work of the multidisciplinary tumour board and integrating available multidisciplinary knowledge, has resulted in an innovative and successful experience. This has made it possible to combine current knowledge and teaching methodology at the level of the actual clinical debate in healthcare centres.

However, both the teaching materials generated and the working methodology developed should be made available to the different subjects that teach oncology, with the aim of unifying diagnostic and therapeutic criteria, avoiding repetition and contradictions and, ultimately, achieving a comprehensive teaching approach to oncology.

This experience could lead to a proposal for educational content in a future elective/optional subject within the study program on Integrative Oncology, taking advantage of possible future revisions of the curriculum, while complementing the current approach, which includes knowledge of oncology through the study of pathology by organs and systems [17].

This project has allowed us to recognise that in degree programmes where oncology is studied in a fragmented manner (and with insufficient knowledge of current work with cancer patients), it is possible to offer students a more comprehensive view of the problem, in line with how it is currently addressed in clinical practice. In any case, it should always be considered a complementary teaching effort that cannot replace the design of a course that addresses the problem of cancer in its entirety. There are well-developed experiences in this regard that could be integrated into medical schools that do not yet have them [4].

## Supplementary Information

Below is the link to the electronic supplementary material.

Supplementary file1 (DOC 1582 KB)

## Data Availability

The data that support the findings of this study are available from the corresponding author upon reasonable request.
