# Supplementary material for: Teaching Oncology in the Medical Degree: Integrating Knowledge and Experience of a Multidisciplinary Tumour Board
Source: J Cancer Educ. 2025 Nov 18;41(5):1021–7. doi: 10.1007/s13187-025-02784-y (PMC13755030; doi:10.1007/s13187-025-02784-y)
Supplement: Supplementary file 1 [file 13187_2025_2784_MOESM1_ESM.doc]

Title: **Teaching oncology in the medical degree: integrating knowledge and experience of a multidisciplinary tumour board**

**Supplemental Material**

Of the 12 real clinical cases included in the seminars, involving patients with tumours locations of intermediate difficulty and complexity, a few are included as examples, following the four-phase learning programme and using the joint report prepared by the relevant experts.

***Phase 1.*** ***Example of a case of Prostate cancer: Clinical history and examination***

*Patient 59 years old.*

*Relatives: Father prostate cancer treated with radiotherapy; mother gastric cancer, sister and first cousin breast cancer,*

*Personal. Married, 1 child. Occupation: businessman.*

*Episodes of back pain, cervical pain due to hernias, Presbycusis.*

*Hypothyroidism: Treatment: Eutirox 75 mcg 50 tbs*

*Allergic to pollen*

*Toxic habits: occasional smoker, moderate drinker.*

*Current History: Patient with minimal discomfort when urination (decreased urine stream and occasional nocturia), who attends his primary care physician in December 2018 who requests PSA (first time) showing elevated values (PSA: 5.4 ng/ml; free/conjugate ratio 9). IPSS is mild. Silodosin^®^ is prescribed for one month and repeat analysis: PSA: 5.96 ng/ml. Digital rectal examination identifies a grade I adenomatous prostate (cT1c). A prostate biopsy is requested and performed with a thick needle on February 2019*

*Commentary: This patient's form of presentation is now common given the accessibility of PSA testing. The validated IPSS scale (International Prostate Symptom Score; with 7 questions and values from 0 to 5) measures the patient's urinary symptomatology (mild <7/moderate 8-19/Severe 20-35).*

*Student questionnaire:*

*1. Do you consider the symptomatology collected in this medical history to be complete?*

*2. Add questions that you consider relevant.*

*3. What additional urinary symptoms would you ask about?*

***Phase 2. Example of a case of Lung cancer case: Diagnosis of certainty and extension.***

*The extension diagnosis includes CT of the chest and abdomen and PET-CT. After the CT result, EBUS is considered the most accessible procedure to sample the tumour.*

***IMAGE Initial CT scan:*** *Chest CT: Solid tumour with rounded contours and heterogeneous density with areas of central necrosis, located in the posterior and anterior segment of the RUL. Its maximum dimensions were approximately 4.5 x 5 x 4.3 cm (AP x T x CC). Surrounding the mass there were areas of increased density in ‘ground glass’ and thickening of the interlobular septa. Right hilar and mediastinal (4R) paratracheal adenopathies of significant size. Assumption: The findings are primarily compatible with bronchogenic carcinoma and associated lymph node involvement.*

***IMAGE Initial PET-CT:*** *PET-CT: Foci of weak-moderate metabolic activity coinciding with adenopathies in mediastinum (stations 2R, 2 -4L and G7). Hypermetabolic focus in right pulmonary hilum, in relation to lymphadenopathies. SULmax 8.85. Hypermetabolic mass in the right lung, over condensation/lung mass in the posterior and anterior segment of the right upper lobe, with an ametabolic centre probably related to necrosis. SULmax 9.79. Mild increase in metabolism in parenchymal infiltrates in the right lower lobe, probably infectious-inflammatory in nature.*

***IMAGE EBUS:*** *Puncture of right lower paratracheal adenopathy (station 4R) guided by linear echo bronchoscopy. In a deeper plane the superior vena cava is visualised. Adenopathy with sonographic signs suggestive of malignancy (well-defined borders, predominantly homogeneous echostructure and absence of central hilar structure).*

***EBUS cytology****: FNA EBUS 4R: Metastasis of adenocarcinoma.*

*After performing a fine needle aspiration puncture guided by Sono bronchoscopy of the 4R mediastinal lymphadenopathy, cytological material is obtained consisting of atypical medium and large non-cohesive cells with prominent nucleoli [lower image in Papanicolaou (left) and in Diff-Quick (right)]. Since there are no cytological characteristics to support a diagnosis, additional immunohistochemical techniques are required to differentiate the histological type of neoplasia. Neoplastic cells show positivity for TTF-1 (upper left image) and negativity for p40 (upper right image).*

*Commentary. The information from immunohistochemical techniques can be key in the diagnosis of lung cancer and many other tumour sites. TTF-1 is a marker of adenocarcinoma and p40 is a marker of squamous carcinoma. In this case it supports the diagnosis of adenocarcinoma.*

*Student questionnaire:*

*1. Cytological material is obtained consisting of atypical discohesive cells with evident nucleoli, which by immunohistochemical techniques is positive for TTF-1 and negative for p40. What is the most likely diagnosis?*

*a. Squamous carcinoma*

*b. Small cell carcinoma*

*c. Adenocarcinoma*

*d. Large cell carcinoma*

*2. If the cytological study showed neoplastic cells in this adenopathy and it was homolateral to the lung lesion, what stage would it be at least? And if it was contralateral to the lesion?*

*3. According to the information available to you, how would you classify the extension of the tumour according to TNM and stage?*

*4. Would any other imaging examination be mandatory?*

***Phase 3. Example of a case of Breast cancer case: MTB and treatment decision***
*Patient diagnosed with Infiltrating Ductal Carcinoma, grade 2, located in the left breast LOQ, cT1cN0M0, Luminal A, presented to the Breast MTB. It was decided to perform tumorectomy plus selective Sentinel Lymph Node Biopsy (SLNB).* *Included are histological images A, B, C, D, and E and pathological anatomy:*

**BSGC: micrometastases *Tumorectomy:*

*Infiltrating ductal carcinoma 20.9 x 20.1 mm.*

*Tumour Histological Grade estimation (Nottinghan Method): Grade 2 (Score: 6/9) [Tubule and gland formation: 3; Nuclear pleomorphism: 2; Mitotic count: 1]*

*Surgical edges free of tumour. Minimum distance 3mm.*

*No perineural or perivascular invasion.*

*Result of immunohistochemical determination of biomarkers:*

*Oestrogen receptors: Intense immunoreactivity (90-100% neoplastic cellularity; +++/+++).*

*Progesterone receptors: Slight immunoreactivity (5-10% neoplastic cellularity; +/+++).*

*HER 2 NEU: Negative*

*Ki 67 (Cell proliferation factor): Overexpression in 5% of proliferating cellularity.*

*Keratin 19: Slight immunoreactivity (70% neoplastic cellularity; ++/+++).*

*E Cadherin: Positive.*

**Stage: pT2 N1mic.*

*Imagen A. Immunohistochemical study of prognostic factors (right down: Ki67, left down: HER2, right up: progesterone receptor, left up: oestrogen receptor)*

*Imagen B. Left breast tumorectomy. Pathological diagnoses of ductal infiltrative carcinoma*

*Commentary*

*The pathologist identifies a lesion with glandular/tubular morphology, with moderate atypia and with a low mitotic index, grading the neoplasm according to the ‘Bloom Richardson Scale’ or ‘Nottingham Score’. It is a combination of nuclear grade, mitotic rate and tubule formation, which describes the appearance of tumour cells under a microscope. The histological grade predicts how aggressive the tumour cells are. This carcinoma is a ductal carcinoma because it forms ducts, tubules or glands, unlike the second most common breast tumour which is lobular carcinoma. It is sometimes very difficult to differentiate and to confirm the ductal origin the E-cadherin immunohistochemical technique is used, which is positive in ductal carcinomas and negative in lobular carcinomas.*

*Student questionnaire (first question):*

*1. What is the system used for breast carcinoma staging?*

*a. Ann Arbor scale*

*b. Bloom Richardson scale*

*c. Milan scale*

*d. Glasgow Scale*

*Imagen C. Ductal infiltrative carcinoma: E-Cadherina +*

*Imagen D. The pathological stage is established based on the pTNM. In this case pT2 as the tumour size is 20.9 x 20.1 mm.*

*The patient was again presented to the breast MTB to assess adjuvant treatment:*

*Adjuvant radiotherapy: complementary to conservative surgery.*

*Hormonal therapy: as it is a tumour with a luminal profile, with positive hormone receptors. An Aromatase Inhibitor is indicated (in this case, Anastrozole).*

*Chemotherapy: this would be indicated, but due to the patient's comorbidities it is ruled out.*

*Image E: Radiotherapy treatment. This image shows the planning of a radiotherapy treatment: i) number of fields; ii) dose distribution, and iii) dose received by the organs at risk (which are the healthy organs close to the treatment field). There are maximum doses that cannot be exceeded in order not to produce toxicity. The treatment administered was: [treatment volume: left breast and axillary lymph node areas I-II-III and supraclavicular (due to lymph node involvement with micro-metastasis and no indication for lymphadenectomy). Dose administered: 40.05 Gy, 15 sessions at 2.67 Gy/session. Daily sessions, 5 sessions/week. Tolerance: grade 1 radiodermatitis in the treatment field.*

*Commentary*

*Radiotherapy treatment is complementary to conservative surgery. In a local treatment with radiation generated in a Linear Electron Accelerator.*

*Student questionnaire (second question):*

*2. In which of the following situations would radiotherapy NOT be indicated?*

*a. mastectomy, pT1N0 tumour*

*b. Conservative surgery, i.e. lumpectomy*

*c. mastectomy, pT3-T4 N0 tumour*

*d. Mastectomy, axillary involvement*

**Supplementary Table 1**: Correspondence between seminar objectives and undergraduate student competencies

| **Recognised outcomes of the Royal College of Radiologists (United Kingdom). rcr.ac.uk *** | **Objectives of Seminar 2** |
| --- | --- |
| (2.1.3) Patient safety: Clinical Practice Guideline | Recognized protocols and pathway for specific tumor location |
| (2.1.4) Dealing with complexity Seeking support | Experts’ discussion presented in every clinical case |
| (2.1.6) Team Working: Multidisciplinary tumour boards | Multidisciplinary role playing |
| 2.2.2. Diagnosis and medical treatment  Typical presentation for common cancers  Importance for sharing decision making | Clinical presentation of case under study  Informs and commentaries of different experts in the case discussion |
| 2.2.4. Describe sources of information for aid decision making  NICE guide | Protocols of the Scientific Society for Innovative methodology |

*Royal college of Physician and Radiology. Undergraduate non-surgical oncology curriculum. September 2020. [www.rcr.uk](http://www.rcr.uk/)

**Final evaluation of the seminars**

*Student questionnaire:*

*1. Did you find the duration of the seminar adequate?*

*2. Did you find the support material provided (clinical case and protocols) useful?*

*3. Do you think that the accompanying images enhance the understanding of the case?*

*4. Do you think that the role of the CMT is clear in the patient care process?*

*5. Do you think that group work is a good option?*

*6. Does the clinical case seem relevant to you?*

*7. Are the procedures used in the diagnosis adequately explained?*

*8. Did the comments help you to understand the case better?*

*9. What about the test reports (CT, PET...)?*

*10. Is the role of the MTB clear in the care process of this case?*

*11. Are the treatment proposals and alternatives clearly presented and explained?*

*12. Is sufficient information provided on the expected outcomes and toxicity?*

*13. Was there any discussion among the members of the group?)*

*14. How would you rate your personal involvement in this seminar?*

*15. How would you rate the value of this seminar for your training?*

***Supplementary Table 2.*** *Results of the student questionnaire (only categories with the highest scores are shown due to the low prevalence of scores <8)*

| Questions | Top three scores provided by students | | | Global percentage of students with high scores (8-10) |
| --- | --- | --- | --- | --- |
|  | 10 | 9 | 8 |  |
| *1. Did you find the duration of the seminar adequate?* | 68.0 | 16.0 | 3.6 | 87.6 |
| *2. Did you find the support material provided (clinical case and protocols) useful?* | 55.4 | 10.7 | 8.9 | 75.0 |
| *3. Do you think that the accompanying images enhance the understanding of the case?* | 52.7 | 12.7 | 18.0 | 83.0 |
| *4. Do you think that the role of the MTB is clear in the patient care process?* | 70.9 | 14.5 | 3.6 | 89.0 |
| *5. Do you think that group work is a good option?* | 69.0 | 10.5 | 11.0 | 90.5 |
| *6. Does the clinical case seem relevant to you?* | 65.0 | 12.0 | 9.0 | 86.0 |
| *7. Are the procedures used in the diagnosis adequately explained?* | 39.0 | 22.0 | 34.0 | 95.0 |
| *8. Did the comments help you to understand the case better?* | 60.0 | 18.0 | 9.0 | 87.0 |
| *9. What about the test reports (CT, PET...)?* | 48.0 | 23.0 | 16.0 | 84.0 |
| *10. Is the role of the MTB clear in the care process of this case?* | 71.0 | 13.0 | 6.0 | 85.0 |
| *11. Are the treatment proposals and alternatives clearly presented and explained?* | 58.0 | 16.0 | 7.5 | 81.5 |
| *12. Is sufficient information provided on the expected outcomes and toxicity?* | 39.0 | 16.0 | 42.0 | 67.0 |
| *13. Was there any discussion among the members of the group?)* | 14.0 | 23.0 | 35.0 | 72.0 |
| *14. How would you rate your personal involvement in this seminar?* | 31.0 | 21.0 | 39.0 | 91.0 |
| *15. How would you rate the value of this seminar for your training?* | 42.9 | 16.0 | 21.0 | 79.9 |

**List of abbreviations**

CAR-T: chimeric antigen receptor cell T

CT: Computed tomography

EBUS: Endo Bronchial Ultra Sound

FNA-EBUS: Fine needle aspiration Endo Bronchial Ultra Sound

Gy: Grey

HER 2 NEU: Human epidermal growth factor receptor 2

LOQ: Lower outer quadrant

TIP: Teaching innovation project

IPSS: International prostatic symptoms score

Ki 67: Cell proliferation factor

RUL: Right upper Lobe

MTB: Multidisciplinary tumour board

PET: Positron emission tomography

PSA: Prostate-specific antigen

pT (pTNM): pathological Tumour

SLNB: Sentinel Lymph Node Biopsy

SULmax. Standard Uptake Lean Body Mass

TNM: Tumour, nodal metastases

TTF-1: Thyroid transcription factor 1
